# Predictive effects of organizational justice on job satisfaction in bus drivers: the moderating effects of role overload and proactive personality

**DOI:** 10.1186/s12889-024-18801-6

**Published:** 2024-05-13

**Authors:** Jingyue Chen, Jiuping Xu, Yi Lu, Wanjie Tang

**Affiliations:** 1 School of Business, Sichuan University, Chengdu, China; 2 West China School of Public Health, Sichuan University, Chengdu, China; 3 Institute of Psychiatry, Psychology & Neuroscience, King’s College London, London, UK

**Keywords:** Procedural justice, Interactional justice, Role overload, Job satisfaction, Bus drivers

## Abstract

**Background:**

There have been few longitudinal studies on Chinese bus drivers and the individual differences in the relationships between organizational justice and job satisfaction. This study examined the organizational justice and job satisfaction in bus drivers and the individual differences in this relationship.

**Methods:**

A two-wave longitudinal study design was employed. A first survey was conducted on 513 Chinese bus drivers in October 2021 that collected socio-demographic information and asked about their perceptions of organizational fairness. A second survey was conducted six months later that asked about role overload and job satisfaction and assessed their proactive personality type. An effect model was then used to explore the moderating effects of role overload and proactive personality type on the relationships between organizational justice and job satisfaction.

**Results:**

Both procedural and interactive justice predicted the bus drivers’ job satisfaction. Proactive personalities and role overload were found to enhance this relationship.

**Conclusions:**

Organizations could benefit from screening at the recruitment stage for drivers with highly proactive personalities. Relevant training for drivers with low proactive personalities could partially improve employee job satisfaction. When viewed from a Chinese collectivist cultural frame, role overload could reflect trust and a sense of belonging, which could enhance job satisfaction. Finally, to improve employee job satisfaction, organizations need to ensure procedural and interactive justice.

**Supplementary Information:**

The online version contains supplementary material available at 10.1186/s12889-024-18801-6.

## Introduction

Satisfied employees generally have good job performances [1]. Satisfied drivers, such as bus drivers, are generally more willing to comply with work safety procedures, which can result in lower accident rates than dissatisfied drivers [2]. There were two deadly traffic accidents in China in 2018 and 2020, which were caused by drivers deliberately driving their buses into a river, both of which resulted in significant casualties [3]. Although it was difficult to determine the reasons for these intentional actions, Chinese transportation enterprise management is seeking to improve their employees’ job satisfaction [4]. The most common reason for employee dissatisfaction is a perception that they are being treated unfairly [5]. However, in the transport industry, bus driver job satisfaction and related organizational justice concerns involve both employee and passenger safety. Chinese bus drivers often have long work hours and night shifts and must deal with passenger relationships, potential passenger conflicts [6], company regulations, and driving safety [7]. Therefore, because of the need to switch between these multiple concerns, they can suffer from role overload [8]. The job demand-control model of work stress [9] indicates that these high demands may affect bus drivers’ job satisfaction [10]. However, as there have been few studies on Chinese bus driver job satisfaction, this study sought to add to the field by exploring bus driver job satisfaction in China.

Though poorly studied in China, job satisfaction and organizational justice, which includes interactive justice and procedural justice, have been widely studied in developed economies for decades [11, 12]. Colquitt’s [13] theory claims that justice and its associated dimensions can have a significant impact on job satisfaction, which consequent studies have confirmed [14]. However, as some results have been inconsistent or even conflicting because of the differences in the sample characteristics and cultures, more research is needed to confirm these relationships. The group value model includes procedural justice as a key antecedent for job satisfaction [15], and the interactions of procedural and interactional justice have been found to predict organizational retaliation behaviors [16]. Employee perceptions of procedural justice have also been found to influence employee job satisfaction in Chinese state-owned enterprises [17]. However, while Rahman et al.’s [18] cross-sectional study did not find any relationship between procedural justice and job satisfaction, interactional justice was observed to have a significant effect on job satisfaction in company executives. Similarly, Sia & Tan [19] found that interactional justice positively affected hotel employee job satisfaction, but procedural justice did not. In contrast, Zainalipour et al. [20] found that procedural and interactive justice were highly positively correlated with job satisfaction in a sample of teachers. While these inconsistent results on the relationship between organizational justice and job satisfaction may have been because of differences in sample sizes, sample properties, cross-sectional designs, or cultural backgrounds, additional job satisfaction evidence is needed on public transport workers in Chinese state-owned companies to better understand the psychological mechanisms between organizational justice and job satisfaction and to provide guidance for state-owned transport enterprise management.

Role overload is when people with limited resources are overburdened with demands, which can result in distraction and work-related stress [21]. Role overload has been negatively associated with job satisfaction in many developed countries [22]. For instance, Pearson’s [23] study on working women found that when their roles were less overloaded, they were more satisfied with their jobs. Lease [24] claimed that role overload was a powerful predictor of many types of strain in academic faculty, which satisfied employees generally have good job performances [25] concluded could lead to negative consequences and significantly undermine psychological well-being. However, Super’s [26] early life span model claimed that multi-role stress could have a positive effect on happiness and satisfaction with life because multiple roles can enhance self-concept and provide a greater number of outlets for a person’s interests, abilities, and values. Therefore, because role overload has been found to affect job satisfaction, the relationship between organizational justice and job satisfaction needs further exploration.

People with proactive personalities are relatively unconstrained by situational forces and environmental changes [27]. Proactive people often show initiative, can identify and solve problems, and can generally persevere and take responsibility for enacting meaningful change. Fuller & Marler’s [28] meta-analytic study found that proactive personalities have positive connections with individual and organization-related outcomes, and Staw and Cohen-Charash’s [29] dispositional job satisfaction model implies that workplace experiences can be influenced by a person’s personality traits. Other studies that confirmed that proactive personalities promote employee job satisfaction speculate that it was because these people have increased self-efficacy [30–32]. Therefore, based on these studies, it is possible the different proactive personality types could influence the relationship between organizational justice and job satisfaction and result in different work performances.

This study makes four research contributions. First, we employ a two-wave longitudinal design on a large, representative sample to determine whether interactive and procedural justice predicts future job satisfaction. As interactive and procedural justice are more prominent in frontline service industry workers [12], understanding the predictive relationships between these two justice types and job satisfaction could enhance organizational justice and strengthen job satisfaction. Second, we examine the moderating effect of role overload on the relationship between interactional or procedural justice and job satisfaction and the associated mechanisms to assess whether targeted programs to increase or decrease role overload could promote job satisfaction. Third, we explore the moderating effect of proactive personality type on the relationship between interactional or procedural justice and job satisfaction to determine whether different proactive personality levels have different effects. Finally, we focus on job satisfaction in Chinese bus drivers in state-owned enterprises to determine methods that could improve their happiness and reduce the risk of public safety incidents resulting from bus driver dissatisfaction.

Gouldner’s [33] reciprocity theory claims that reciprocity is vital to the maintenance of stable social systems, such as organizations, and interpersonal relationships. When people put all their energy, enthusiasm, and labor into providing services for others, they naturally expect to get considerable returns. Generally speaking, in the service industry, management, passengers, and front-line drivers form pairwise liaison relationships, with the front-line employees being the important relationship nodes [34] However, this interpersonal interactive balance often breaks down when front-line employees are not rewarded for their efforts. Although interactional justice has typically been used to assess supervisor fairness, many front-line workers may feel a sense of injustice when treated badly by customers and when they have to use more physical and psychological resources at work than is required by their salary and benefit levels [35]. Therefore, service interaction unfairness could also lead to front-line employee job dissatisfaction [36].

Organizational support theory [37] posits that procedural fairness fosters a stronger sense of organizational support compared to other fairness dimensions, consequently bolstering job satisfaction. A meta-analysis study revealed a significant correlation between procedural justice and job satisfaction, with a weighted mean r of 0.47 [38]. Procedural justice not only elevates support expectations but also fulfills socioemotional needs [37, 39], establishing it as a reliable predictor of job satisfaction [40]. Recent studies have also found that procedural fairness directly affects job satisfaction among cement enterprise employees [41]. However, other studies have shown that procedural justice has no effect on job satisfaction among bank employees [42]. Therefore, more evidence is needed to explore the relationship between procedural justice and job satisfaction, for example, in the group of bus drivers. Because bus drivers in China suffer from driver-passenger communicative stress [3], any lack of interaction fairness could seriously affect their job satisfaction.

In sum, this study expands on previous research to explore how interactive and procedural justice can predict front-line employee job satisfaction. Based on this discussion, the following hypotheses are proposed.

### Hypothesis 1a

The perceived interactive justice of bus drivers positively predicts their job satisfaction.

### Hypothesis 1b

The perceived procedural justice of bus drivers positively predicts job satisfaction.

Proactive personality tests assess the propensity to engage in proactive behaviors to affect environmental change [27]. There is a dynamic interrelationship between people and their environment, which is characterized by reciprocal causal links [43]. Bandura’s [44] social cognition theory claims that people, environments, and behaviors constantly influence each other and that people can actively create and change their environments and others’ behaviors to better adapt to the environment. Therefore, people with proactive personalities can actively challenge their environment rather than passively adapt to it. Crant [45] proposed an integrated proactive personality model for organizational behaviors, which suggested that a critical determinant of organizational success is when employees with proactive personalities take the initiative to change their environments and processes at work to positively impact their work and careers. Social capital theory also suggests that proactive employees can gain performance benefits by developing social networks that provide them with the resources and latitude to pursue high-level initiatives [46]. Therefore, people with proactive personalities are more able to change their work environments and situations and adjust their thinking and emotions to positively adapt to any unfair work situation, which, in turn, could increase their job satisfaction. However, people with low proactive personalities may passively tolerate their work environment. Therefore, job satisfaction could be affected by the different proactive personality attitudes toward procedural and interactive justice. Based on this discussion, we propose the following hypotheses.

### Hypothesis 2a

A proactive personality positively moderates the relationship between procedural justice and job satisfaction.

### Hypothesis 2b

A proactive personality positively moderates the relationship between interactional justice and job satisfaction.

Role overload is connected to the role stress people feel when they are cognitively overtaxed because of excessive time pressures, commitments, or responsibilities [47]. Role overload, which occurs when there are too many role demands given the time and resources available [25] and is often found in customer-oriented organizations with scarce resources [48], can cause an increase in burnout and job stress [49] and employee retention and productivity problems [50]. People who feel role overload can have significantly lower levels of psychological well-being, such as job dissatisfaction, compared to those who do not experience these feelings. However, the stress-management model of job strain [9] (Karasek Jr, 1979) posits that high job demands (role overload) are not necessarily harmful; however, when these demands are accompanied by low decision latitude, psychological strain can result. Studies have found positive relationships between high job demands, indices of strain, and negative mental health [51–54]. Role overload was also found to moderate the direct effects of self-efficacy and work performance in a sample of service industry employees [55]. Given the potential interrelationships between job overload, perceived organizational justice, and job satisfaction, it is possible that different role overload levels could attenuate the influence of procedural and interactive justice on job satisfaction. Give the above discussion, the following hypotheses are proposed.

### Hypothesis 3a

Role overload negatively moderates the relationship between procedural justice and job satisfaction.

### Hypothesis 3b

Role overload negatively moderates the relationship between interactional justice and job satisfaction.

## Methods

### Data and sample

This two-wave longitudinal study was approved by the Research Ethics Committee of Sichuan University, approval number K2021027. This study adopted a convenience sampling method through negotiation and cooperation with the bus companies. Two paper-based surveys were conducted approximately six months apart on bus drivers in the Shuangliu District (only one bus company in this area), an administrative area of Chengdu in Southwest China. China’s 2020 census data revealed that Shuangliu District has a resident population of 1.465 million and an area of 466 square kilometers. The bus companies were responsible for recruiting the drivers for the surveys, the administration of which took place at specially organized meetings.

The surveys were handed out and explained at these meetings by seven psychology/business administration Master’s or Ph.D. students, and the drivers were asked to complete them within two weeks. Each driver who completed the survey was given two towels. The first survey was conducted in the last two weeks of October 2021, and the second survey was completed in the last two weeks of March 2022. Each survey took about 10 min. Of the 585 district bus drivers, the 540 drivers who participated in the first survey were invited to participate in the second survey. Finally, 515 drivers completed both surveys.

### Measures

The first survey asked about the bus drivers’ perceptions of procedural and interactive fairness and collected data on their sleep duration, nap times, and other sociodemographic variables, and the second survey collected data on role overload, proactive personality type, and job satisfaction.

#### Front-line employee sense of justice

Colquitt’s organizational justice scale [56], which examines front-line employee perceptions of fairness, was adapted to better fit Chinese front-line employee cultural characteristics [12]. The scale, which primarily measures procedural justice (nine items) and interactive justice (three items), uses a 7-point Likert scale from 1 = never to 7 = always. Sample items include *Customers communicate with me in a timely manner* and *I am treated with dignity by customers*. In this study, Cronbach’s alphas for procedural and interactive were respectively 0.901 and 0.783.

#### Role overload

To better conform to the characteristics of Chinese culture [57], the Role Overload Scale (three items) was adapted from three role stress and role load scales [58–60]. The three items were: *There are too many things required of me at work; There are too many things expected of me by my superiors;* and *I have too much work for me alone*. In this study, Cronbach’s alpha for this scale was 0.839.

#### Proactive personality

The Chinese version of the Proactive Personality Scale (ten items) [61] was adapted from two other scales [27, 62]. Sample items are: *I’m willing to stand up for my ideas even when others disagree* and I*’m good at spotting opportunities*. The scale uses a five-point scale ranging from strongly disagree = 1 to strongly agree = 5. Cronbach’s alpha for this scale was 0.803.

#### Job satisfaction

The job satisfaction scale was based on previous job satisfaction scales and was also adapted to consider Chinese culture [63, 64]. This five-item scale focuses on satisfaction with the work environment and salary and uses a five-point Likert scale. Sample items include: *I am satisfied with my current salary* and *I am satisfied with the interpersonal interaction between other people and me in the work environment*. Cronbach’s alpha for this scale was 0.890.

### Control variables

As age [65], gender [66], years of service [67], and sleep quality [68] can impact job satisfaction, age, gender, length of service, sleep duration at night, and nap time were the control variables. Length of service was divided into five stages: 1–5 years, 6–10 years, 11–15 years, 16–20 years, and more than 21 years, sleep duration was measured in hours, and nap time was measured in minutes.

### Data analysis

IBM SPSS 23.0 statistical software was used for the data analyses. First, the descriptive statistics were calculated: age, gender, length of service, sleep duration, and nap duration. Second, Pearson’s correlation was used to determine the correlations between the variables. Third, stepwise regression was employed to assess the relationships between the major variables and job satisfaction; the first step involved inputting the social demographic variables(age, gender, and length of service), the second step involved inputting the interactive and procedural justice variables, the third step involved inputting the role load variables, and the fourth step involved inputting the proactive personality variables. Finally, to verify the hypotheses, Hayes’s model 1 PROCESS macro of SPSS was adopted to calculate the predictive relationships between procedural justice, interactive justice, job satisfaction, and the moderating effects of role overload and proactive personality.

## Results

### Descriptive statistics

Of the 515 completed surveys, two were removed for quality control purposes, such as choosing option A for all questions; therefore, the data from 513 participants were included in the statistical analysis. The descriptive statistical results are shown in Table 1. Of the 513 participants: 497 (96.9%) were male at T1; 42 were 28–35 years old, 312 were 36–50, and 149 were 50–59; and, 208 had 1–5 years of service, 184 had 6–10 years of service, 78 had 11–15 years of service, 29 had 16–20 years of service, and 14 had more than 21 years of service.

**Table 1:** Bus drivers’ demographic variables at Time 1(*n* = 513)

Variables	*n*	Prevalence (%)
Total	(*n* = 513)	100
Gender		
Male(*n* = 497) Female(*n* = 16)	497 16	96.9 3.1
Age (yr)		
25–35 36–50 50–59	42 312 149	8.2 62.8 29.0
Length of service		
1–5	208	40.5
6–10	184	35.9
11–15	78	15.2
16–20	29	5.7
21–41	14	2.7
Sleep duration (hour)		
≤ 5	6	1.2
6–7	76	14.8
7–8	186	36.3
8–9	229	44.6
9–11	16	3.1
Nap time (minute)		
0 10–20 21–30 31–60	38 210 177 88	7.4 40.9 34.5 17.2

### Bivariate correlation analysis

The correlation results are shown in Table 2. The T1 procedural justice and interactive justice correlations with T2 job satisfaction were between 0.33 and 0.35. The T2 proactive personality was positively correlated with T2 role overload and T2 job satisfaction at respective correlations of *r* = 0.56 and *r* = 0.63. Role overload was moderately correlated with job satisfaction (*r* = 0.28).

**Table 2:** Correlations for the main study variables (*N* = 513)

Variable	M	S	1	2	3	4	5	6	7	8	9	10
1. Age	46.77	6.48	1									
2. Gender	n/a	n/a	-0.09	1								
3. Length of service	8.23	5.46	0.38**	-0.03	1							
4. Nap time	n/a	n/a	0.07	-0.05	0.03	1						
5. Sleep duration	7.41	0.85	-0.06	-0.09	0.01	0.06	1					
6. T1 Interactional Justice	32.66	9.05	-0.05	0.01	0.02	-0.02	-0.01	1				
7. T1 Procedural Justice	10.94	3.85	-0.05	-0.03	0.04	0.06	0.01	0.63**	1			
8. T2 Proactive personality	32.73	7.59	0.08	0.01	0.05	-0.05	0.04	0.25**	0.26**	1		
9. T2 Role overload	9.45	3.28	0.11*	-0.08	0.05	-0.02	-0.02	0.17**	0.14**	0.56**	1	
10. T2 Job satisfaction	19.18	4.75	-0.04	0.04	-0.01	0.06	0.04	0.35**	0.33**	0.63**	0.28**	1

*p < 0.05, **p < 0.01; n/a, not applicable; T1, time 1; T2, time 2

### Predictive analysis and moderation effect analysis

**Fig. 1:**
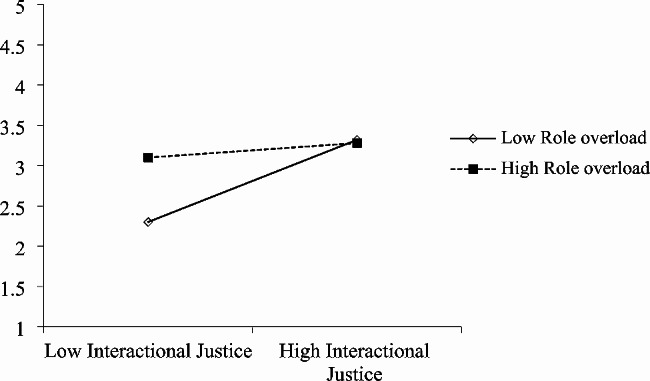
Moderating effect of T2 role overload on T1 interactional justice predicting T2 job satisfaction

**Fig. 2:**
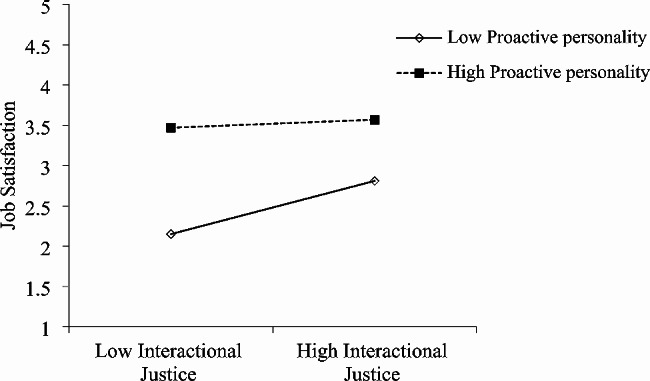
Moderating effect of T2 proactive personality on T1 interactional justice predicting T2 job satisfaction

**Fig. 3:**
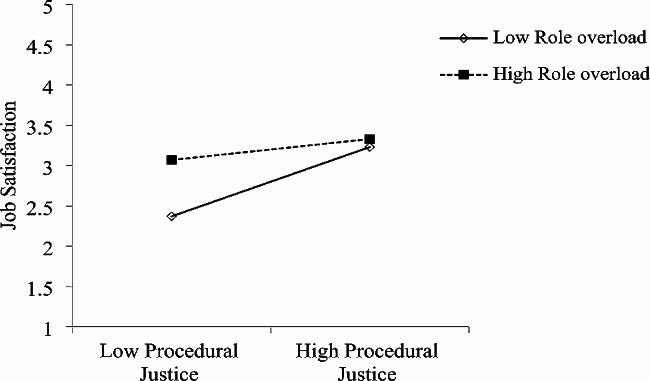
Moderating effect of T2 role overload on T1 procedural justice predicting T2 job satisfaction

**Fig. 4:**
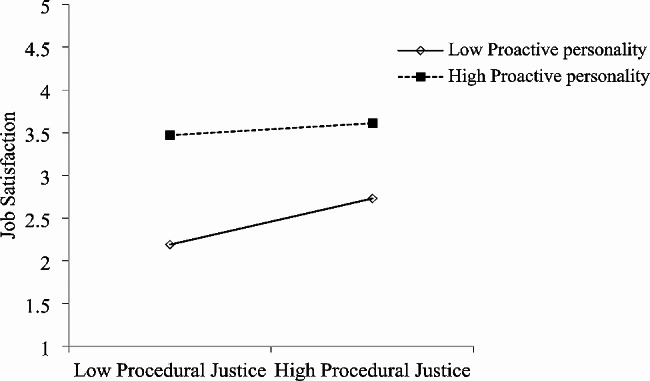
Moderating effect of T2 proactive personality on T1 procedural justice predicting T2 job satisfaction

Both interactive justice and procedural justice at T1 were found to predict future job satisfaction at T2. As shown in Fig. 1; Table 3, T2 role overload moderated the association between T1 interactional justice and later T2 job satisfaction (b = 0.21, *p* < 0.001; R^2^ = 0.23, F = 50.69, *p* < 0.001). As shown in Table 4; Fig. 2, T2 proactive personality moderated the association between T1 interactional justice and later T2 job satisfaction (b = -0.14, *p* < 0.001; R^2^ = 0.47, F = 147.95, *p* < 0.001). Similarly, T2 role overload moderated the association between T1 procedural justice and later T2 job satisfaction (b = -0.15, *p* < 0.001; R^2^ = 0.19, F = 40.96, *p* < 0.001) (Table 5; Fig. 3), and T2 proactive personality moderated the association between T1 procedural justice and later T2 job satisfaction (b = -0.10, *p* < 0.001; R^2^ = 0.44, F = 132.25, *p* < 0.001) (Table 6; Fig. 4).

### Regression analysis

The supplementary files in Table 7 highlight the stepwise regression findings. In step 1, T1 interactional justice (***β*** = 0.231, *p* < 0.001) and procedural justice (***β*** = 0.183, *p* < 0.001) added significance (ΔR^2^ = 0.141, *p* < 0.001). In step 2, the addition of T2 role overload (***β*** = 0.215, *p* < 0.001) showed a significant association with T2 job satisfaction (ΔR^2^ = 0.056, *p* < 0.001), T1 interactional justice (***β*** = 0.22, *p* < 0.001), and procedural justice (***β*** = 0.175, *p* < 0.001) remained significant with T2 job satisfaction. In step 3, the addition of T2 proactive personality (***β*** = 0.637, *p* < 0.001) showed significant association with T2 job satisfaction (ΔR^2^ = 0.263, *p* < 0.001), T1 interactional justice (***β*** = 0.149, *p* < 0.001), and procedural justice (***β*** = 0.078, *p* < 0.05), and T2 role overload (***β*** = 0.118, *p* < 0.01) remained significant with T2 job satisfaction. In step 4, the addition of their interaction showed only T1 interactional justice (***β*** = 0.874, *p* < 0.001), T2 Proactive personality (***β*** = 1.072, *p* < 0.001) and T1 Interactional Justice× T2 Proactive personality (***β***=-1.074, *p* < 0.001) remain significantly with T2 job satisfaction.

**Table 3:** T2 role overload as a moderator for T1 interactional justice predicting T2 job satisfaction in bus drivers (*N* = 513)

Predictors	Model 1 (T2 Job satisfaction)				Model 2 (T2 Job satisfaction)				
	β		t			β		t	
T2 Role overload	0.28		6.68^***^			0.19		4.62^***^	
T1 Interactional Justice						0.30		7.50^***^	
T1 Interactional Justice × T2 Role overload						-0.21		-6.19^***^	
R^2^	0.08					0.23			
F	44.63^***^					50.69^***^			

*p < 0.05; **p < 0.01; ***p < 0.001. T1, time 1; T2, time 2

**Table 4:** T2 proactive personality as a moderator for T1 interactional justice predicting T2 job satisfaction in bus drivers (*N* = 513)

Predictors	Model 1 (T2 Job satisfaction)				Model 2 (T2 Job satisfaction)				
	β		t			β		t	
T2 Proactive personality	0.63		18.33^***^			0.52		14.54^***^	
T1 Interactional Justice						0.19		5.67^***^	
T1 Interactional Justice× T2 Proactive personality						-0.14		-5.35^***^	
R^2^	0.40					0.47			
F	336.08^***^					147.95^***^			

***p < 0.001. T1, time 1; T2, time 2

**Table 5:** T2 Role overload as a moderator for T1 procedural justice predicting T2 job satisfaction in bus drivers (*N* = 513)

Predictors	Model 1 (T2 Job satisfaction)				Model 2 (T2 Job satisfaction)				
	β		t			β		t	
T2 Role overload	0.28		6.68^***^			0.20		4.88^***^	
T1 Procedural Justice						0.28		7.04^***^	
T1 Procedural Justice × T2 Role overload						-0.15		-4.27^***^	
R^2^	0.08					0.19			
F	44.63^***^					40.96^***^			

**p < 0.01; ***p < 0.001. T1, time 1; T2, time 2

**Table 6:** Proactive personality as a moderator for T1 procedural justice predicting T2 job satisfaction in bus drivers (*N* = 513)

Predictors	Model 1 (T2 Job satisfaction)				Model 2 (T2 Job satisfaction)			
	β		t		β		t	
T2 Proactive personality	0.63		18.33^***^		0.54		14.43^***^	
T1 Procedural Justice					0.17		4.84^***^	
T1 Procedural Justice× T2 Proactive personality					-0.10		-3.25^**^	
R^2^	0.40				0.44			
F	336.08^***^				132.25^***^			

**p < 0.01; ***p < 0.001. T1, time 1; T2, time 2

## Discussion

Bus drivers satisfied with their jobs have greater company loyalty, better job performances, and pay greater attention to passenger safety [2, 69]. Using proactive personality and role overload as moderators for the links between organizational justice and job satisfaction, we explored the predictive associations between procedural justice, interactional justice, and job satisfaction. Our data confirms that both procedural justice and interactional justice can predict job satisfaction, and when age, gender, and length of service are controlled, proactive personality and/or role overload moderates this effect.

Many organizational and individual-level factors can affect job satisfaction [70]. Colquitt’s [13] theory posits that different fairness dimensions can have varying effects on job satisfaction. This study expands on previous studies by demonstrating the important predictive effect of organizational justice (procedural justice and interactive justice) on the job satisfaction of Chinese bus drivers and verifies the differential effects of role overload and proactive personality levels on this job satisfaction effect.

As hypothesized, procedural justice predicts later bus driver job satisfaction, which is consistent with Chinese evidence from both state-owned and private businesses [17], Middle Eastern transportation companies [71], German factory employees [72], all of which conclude that the employees’ perception of justice related to work procedures significantly affects job satisfaction. However, another study on pharmaceutical company employees in Bangladesh concludes that procedural justice has no relationship with job satisfaction [18]. This difference may be because of differences in business type and the sample.

This current study extends these previous cross-sectional studies by longitudinally finding that job satisfaction in bus drivers can be accurately predicted from their perceived procedural justice. Organizational support theory [37] claims that as employees view procedural justice as an organizational responsibility, it has a strong relationship with perceived organizational support, self-enhancement, and the consequences of these, such as job satisfaction.

The findings from this study have practical implications, that is, when bus management decision-making and procedures are perceived to be fair, bus drivers will have improved job satisfaction; however, if they feel that the company decision-making processes are unfair when dealing with customer disputes, they will become more dissatisfied with their work. Therefore, to avoid such situations, management needs to allow drivers to truly express their views and feelings when they feel they are being treated with disrespect by customers and be given the option to participate in formulating subsequent amendments to passenger service regulations and the corresponding ethical and moral norms.

This study also confirms the research hypothesis that interactional justice predicts bus driver job satisfaction, which is consistent with a study on bank workers from Pakistan that found that the fairness of interactions with customers is associated with job satisfaction [14]. In another cross-sectional study, nurses’ perceptions of fairness in their interactions with patients were found to be strongly associated with job satisfaction [73]. The study results are also similar to a study on white-collar workers that found interactive fairness to be an antecedent for job satisfaction [74], a transportation industry worker study [71], and an upscale international hotel study [75].

These results can also be explained by the reciprocity of human society theory [33] and Blau’s social exchange theory [76], both of which suggest that interpersonal communication requires equal obligation relationships between the two parties, and if one party has energy and enthusiasm, they hope to be treated and rewarded fairly. Therefore, if the interpersonal communication working relationships between bus drivers and passengers are fair, the bus drivers feel equal, respected, and satisfied with their work; otherwise, they feel dissatisfied with their work. This finding implies that when passengers openly and clearly communicate with bus drivers in a polite, dignified, and respectful manner, bus drivers will have higher job satisfaction, and also suggests that bus drivers should be better trained in good communication skills as this could result in better passenger feedback and a sense of fairness in communication, which would also enhance job satisfaction.

Another important finding was the intergroup effect of proactive personality differences on the relationship between perceived fairness and job satisfaction. Bus drivers with high proactive personalities had higher job satisfaction regardless of their perceived fairness, which was partly consistent with previous studies that found that proactive personalities are positively correlated with job satisfaction [77, 78], even over time [30]. The moderating effect of the proactive personality could be related to the steadfast orientation of the proactive personality trait to be supportive in difficult times [79]. These results could also be explained by Bandura’s social adaptation theory [44], that is, drivers with highly proactive personalities can create more favorable interpersonal environments and can better adapt to the environment, which leads to higher job satisfaction even in adverse external environments.

Proactive personalities have also been found to be closely related to self-efficacy and work engagement, which can also lead to increased job satisfaction [78]. However, the procedural justice and interactional fairness perceptions in people with low proactive personalities are more sensitive in the relationship with job satisfaction. If the perceived fairness is high, the job satisfaction is high, but if the perceived fairness is low, the job satisfaction is low. The research findings also imply that if drivers with highly proactive personalities are screened as part of the recruitment process, they could have more stable job satisfaction because of their ability to positively adapt to the environment. Drivers with low proactive personalities, however, could be positively shaped and influenced through training programs to increase their job satisfaction.

It was also found that role overload could be another potential psychological mechanism in the relationship between perceived justice and job satisfaction. Somewhat different than the hypothesis, role overload was found to enhance rather than attenuate the effect of procedural or interactive justice on job satisfaction. Contrary to our study results, most studies have found that role overload negatively affects job satisfaction. For instance, Kacmar et al. [80] found that role overload attenuated the relationship between resilience and family-work enrichment, with the relationship being found to be weaker when role overload was high. Role overload has also been found to negatively affect the job satisfaction of full-time employees [81–83]. The contrary results found in this study could be explained by the stress-management model of job strain [9], which posits that high job demands (role overload) are not harmful. For example, Janssen [84] found a positive relationship between job demands and innovative work behavior when employees perceived work-reward fairness. The Yerkes-Dodson law [85] claims that greater role stress within some tolerable limits can lead to better performances; therefore, this aspect needs further exploration as this study found that perceived justice resulted in greater job satisfaction, especially when there were more role demands, which may have been because of the bus drivers’ increased sense of being needed and valued. However, these suppositions require further research to explore the related mechanisms.

### Limitation

Despite the longitudinal study design and the important contributions, there were some limitations to this study. First, the collectivist Chinese characteristics and the Chinese metropolitan sample may not be representative of other areas in China or other developing countries. Therefore, a cross-regional comparison of China and a more representative sample selected at random or a cross-cultural comparison may help address this limitation. Second, as this study used a self-assessment questionnaire, self-report subjectivity may have affected the reliability and validity of the variables. Therefore, a combination of objective tools, such as brain imaging, electrophysiological methods, or more in-depth interviews with drivers and management may address this limitation. Third, although we consider the temporal order of the variables, it may be better to include all variables at both time points. Finally, when organizational justice was assessed, only procedural and interactive justice were included in the communication and interactions between front-line bus drivers and passengers; therefore, as distributional justice was not considered, this needs to be explored in future research.

## Conclusion

In summary, based on the longitudinal study design, it was found that both the bus drivers’ perceived procedural and interactive fairness predicts future job satisfaction. As this also tested the moderating effects of role overload and proactive personality on the links between organizational justice (procedural and interactive justice) and job satisfaction for front-line employees, it has important theoretical and practical implications and makes an important contribution to existing literature. Past research found that employees with high perceived procedural or interactive fairness have high job satisfaction; however, most of these studies have been cross-sectional studies rather than longitudinal studies or have attempted to explore the effects of individual differences. Given these research gaps, this study reveals that role overload and a proactive personality both enhance the tendency for procedural and interactive perceptions of fairness to promote job satisfaction.

This study, therefore, has significant practical value. Enterprises need to consider both organizational and employee fairness when developing procedures to resolve passenger and driver conflicts, and when there are conflicts or interactions, drivers should be given communication skills through standardized training to ensure better passenger feedback and perceived fair treatment. Organizations would benefit from hiring more proactive individuals because they have higher job satisfaction than those with less proactive personalities. At the same time, organizations can also train individuals with poor proactive personalities to experience greater job satisfaction by being more proactive. In addition, giving employees greater role stress within an appropriate scope may make employees perceive greater trust in the organization [86], which would also enhance job satisfaction.

### Electronic supplementary material

Below is the link to the electronic supplementary material.

Supplementary Material 1

## Data Availability

No datasets were generated or analysed during the current study.
