# Supplementary material for: Predictive effects of organizational justice on job satisfaction in bus drivers: the moderating effects of role overload and proactive personality
Source: BMC Public Health. 2024 May 13;24:1294. doi: 10.1186/s12889-024-18801-6 (PMC11089838; doi:10.1186/s12889-024-18801-6)
Supplement: Supplementary file 1 — Supplementary Material 1 [file 12889_2024_18801_MOESM1_ESM.docx]

Table 7. Linear regression for job satisfaction on organizational justice, role overload, proactive personality and their interaction viariables (n = 513)

| Independent variable | Step 1:  R^2^=.141 | | Step 2:  R^2^= .187 | | Step 3:  R^2^= .450 | | Step 4:  R^2^= .484 |
| --- | --- | --- | --- | --- | --- | --- | --- |
|  | Beta |  | Beta |  | Beta |  | Beta |
| T1 Interactional Justice | .231*** |  | .220*** |  | .149*** |  | .874*** |
| T1 Procedural Justice | .183*** |  | .175*** |  | .078* |  | -.106 |
| T2 Role overload |  |  | .215*** |  | .118** |  | -.110 |
| T2 Proactive personality |  |  |  |  | .637*** |  | 1.071*** |
| T1 Interactional Justice×  T2 Proactive personality |  |  |  |  |  |  | -1.074*** |
| T1 Procedural Justice × T2 Proactive personality |  |  |  |  |  |  | .323 |
| T1 Interactional Justice× T2 Role overload |  |  |  |  |  |  | .057 |
| T1 Procedural Justice × T2 Role overload |  |  |  |  |  |  | -.104 |

*p<0.05 ; **p<0.01; ***p<0.001 ; T1, time 1; T2, time 2.
